# Supplementary material for: Vitamin D Modified DSS-Induced Colitis in Mice via STING Signaling Pathway
Source: Biology (Basel). 2025 Jun 18;14(6):715. doi: 10.3390/biology14060715 (PMC12190092; doi:10.3390/biology14060715)
Supplement: Supplementary file 1 [file biology-14-00715-s001.zip › Supplementary Materials S1: 16s-report of gut microbiota/Groups/group.html]

谷禾信息 结题报告


### 6 分组分析结果

##### 6.1 分组元数据统计

对分组样本及其元数据进行统计。

结果文件是:group\_compare.html

### 帮助文档
